# Surgical treatment of benign osteolytic lesions in the femoral head and neck: a systematic review

**DOI:** 10.1186/s12891-021-04442-y

**Published:** 2021-06-16

**Authors:** Jingtian Shi, Zhiqing Zhao, Taiqiang Yan, Wei Guo, Rongli Yang, Xiaodong Tang, Huayi Qu, Sen Dong

**Affiliations:** Musculoskeletal Tumor Center, Peking University People’s Hospital, Beijing, 100044 China

**Keywords:** Femoral head and neck, Benign, Osteolytic lesions, Curettage, Outcomes

## Abstract

**Background and objectives:**

Treatment of benign osteolytic lesions in the femoral head and neck can be extremely challenging, particularly in children with open physis or for aggressive tumors with pathological fracture. There remains the difficult management decision as to whether to perform complete excision of the involved area or only curettage. Moreover, there is no agreed consensus on the optimal approach to lesion access when performing curettage, which included the transcervical, open and direct approach. The current systematic review aims to provide guidance for selection of surgical methods in clinical practice by comparing the advantages and drawbacks of different procedures.

**Methods:**

A comprehensive literature search of PubMed, Embase and Web of Science databases were executed for human studies restricted to the English language. The search was filtered to include studies published from January 1980 to January 2020.

**Results:**

A total of 33 articles including 274 patients were enrolled in the final analysis. The most common diagnosis was chondroblastoma (CBT) (104, 38.0%), followed by giant cell tumor (GCT) (56, 20.4%). There were 57 (20.8%) patients with pathological fracture. Intralesional curettage was performed in 257 (93.8%) patients with the local recurrence of 12.5% at the mean follow-up of 51.5 months. The patients who were presented with open physis or curetted via transcervical approach developed higher local recurrence in patients with CBT (*P* < 0.001). The local recurrence rate of GCT is 33.3% after curettage, while 8 of 9 (88.9%) patients with fracture were treated successfully with joint preservation. Two of 45 (4.4%) patients developed avascular necrosis (AVN) of femoral head after surgical hip dislocation. The reported Musculoskeletal Tumor Society (MSTS) Score was comparable among patients with different approaches to curettage.

**Conclusion:**

The majority of benign osteolytic lesions in the femoral head and neck can be treated with intralesional curettage with acceptable local tumor control and satisfactory function. The incidence of local recurrence might be decreased dramatically for lesion access under direct visualization. The native joint maintenance could be achieved even in patients with aggressive lesions presenting pathological fracture.

**Supplementary Information:**

The online version contains supplementary material available at 10.1186/s12891-021-04442-y.

## Introduction

Benign osteolytic lesions throughout the skeleton encompass a group of neoplastic or tumor-like conditions, such as chondroblastoma (CBT), giant cell tumor (GCT) and others. Proximal femur can be a common site where the femoral head and neck is less frequently involved than the trochanteric region. Patients often seek consultation for help with mechanical symptoms of pain related to activity and difficulty in ambulating due to compromise of osseous integrity [1]. Different treatment protocols have been described in previous literatures. Considering the normal life expectancy of these patients, intralesional curettage is the primary treatment of choice for these lesions [2–4]. Wide resection following hemi or total hip replacement is not recommended as initial treatment while may be inevitable in more complicated situations [5]. Moreover, minimal invasive technique such as radiofrequency ablation (RFA) has also been reported [6–10].

With regard to intralesional curettage, there are three options for lesion access [11]. The first is transcervical approach, via a bone tunnel through the femoral neck from the lateral cortex of proximal femur [11, 12]. The second is open approach, via an open window of the femoral neck following capsulotomy [13, 14]. The third is direct approach, via the surface of femoral head after surgical hip dislocation [4, 15–17]. The latter two were designed to process the procedure under direct visualization. To minimize the residual tumor tissue, use of high-speed burr or other adjuvant therapies after curettage is reported to play a role [11, 18]. Filling the cavity caused by curettage is another indispensable step, commonly with bone graft or cement and sometimes, combined with internal fixation devices [3, 13, 19]. The proper selection of the surgical approach is a cornerstone for exposure of any neoplastic lesion. This is particularly essential for lesions in the femoral head and neck, which is known with anatomical constraints [6].

The literatures on benign osteolytic lesions in the femoral head and neck are limited so that some problems remain unanswered such as the factors influencing the recurrence and the postoperative complications. This systematic review aims to ascertain the following questions: (1) what are the common benign osteolytic lesions of femoral head and neck? (2) what factors act in the local recurrence after intralesional curettage? (3) is pathological fracture caused by aggressive tumors treated successfully by joint preserving procedure? (4) does direct approach lead to higher incidence of femoral head avascular necrosis (AVN)? (5) do these approaches to lesion access result in the difference of functional outcome? To answer the above-mentioned questions, a pooled analysis was performed utilizing the data of all patients in selected studies.

## Methods

### Search strategy and inclusion criteria

The study design included a review of studies published from 1980 January to January 2020 on PubMed, Embase and Web of Science databases. The terms employed for search included: (((((femoral head) OR femoral neck) OR proximal femur) AND bone tumor) AND surgery). A set of inclusion and exclusion methodology was formed based on preferred reporting items for systematic reviews and meta-analyses (PRISMA) guidelines [20]. The methodological quality of each studies was evaluated by the Methodological Index for non-Randomized Studies (MINORS), which evaluated 8 items for noncomparative studies with a maximum score of 16 [21]. A score of 12 or higher was considered as “high”, 9–11 was considered as “moderate” and 8 or less was considered as “low” quality [22]. The articles which met the eligibility were rated by the same two authors and when the discrepancies occurred, a discussion was warranted for final decision. The studies included in the final analysis fulfilling the following eligibility criteria: (1) the full text was available in English and restricted to human study. (2) the lesions located merely at the femoral head and neck were osteolytic and histologically confirmed as benign. (3) patients were treated with surgical management. The exclusion criteria were articles describing: (1) lesions in the trochanteric region even with femoral head and neck extension. (2) primary malignant or metastatic tumors. (3) synovial lesions such as PVNS and synovial chondromatosis. (4) AVN of femoral head, osteomyelitis or developmental disorder. Due to the rarity of lesions in femoral head and neck, we also included case series more than 3 patients. Articles reporting a cohort of patients with multiple sites of lesion were included only when separate data for patients with benign osteolytic lesions in the femoral head and neck could be gathered. Case reports, reviews, letters, comments, conference abstracts, editorials were excluded.

### Study selection and assessment of quality

The search protocol and article selection were completed independently by two authors (SJT and ZZQ) to identify potentially relevant studies. The references of the articles that met inclusion criteria after screening were then reviewed manually to find out additional studies not captured in the initial search. The articles utilized for the final analysis were determined by the senior author (YTQ). Figure S1 is a flowchart showing the steps to select the studies for the current systematic review.

### Data distraction

The patient characteristics reported from each study included number of patients who met the inclusion criteria, age, gender, diagnosis, location, status of growth plate, pathological fracture and maximum diameter of the lesion. The intraoperative information gathered included the type of surgery, method used for lesion access and employment of adjuvants as well as void filler for curettage group. Postoperative results included the duration of follow-up, incidence of recurrence and time to recurrence, complications (growth disturbance in skeletally immature with CBT, AVN of femoral head and degenerative changes after hip dislocation and other nonspecific sequelae such as infection, mechanical failure, deformity and so on), and Musculoskeletal Tumor Society (MSTS) score, which is commonly utilized to evaluate patient outcomes following musculoskeletal oncological surgery.

### Statistical analysis

Two authors (SJT and YTQ) analyzed the data. Microsoft Excel® (Microsoft Corporation, Redmond, WA) was utilized to calculate averages, median and range. The SPSS Version 20.0 (IBM Corp, Armonk, New York, USA) was used for statistical analysis. Cohort differences were analyzed by non-parametric test for variables which were presented with median and range. Chi-square test (Fisher’s Exact tests) for incidence of postoperative complications and recurrence which were presented with frequency with percentage. A *P*-value less than 0.05 was considered statistically significant. Graphical representations of data, including box-plots, pie-graphs and bar-graphs, were created using Stata Version 16® (Stata Corp, College Station, TX).

## Results

### Study and patient characteristics

**Fig. 1:**
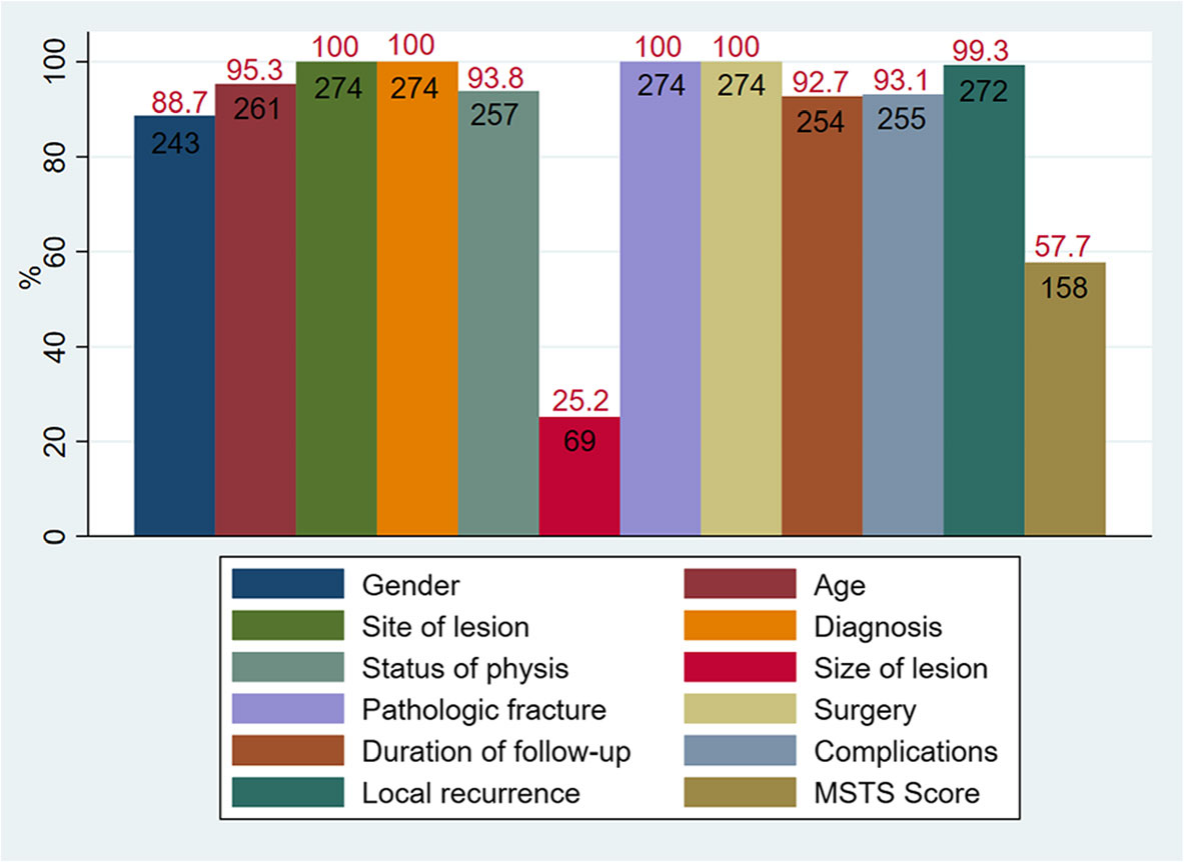
The bar graph shows percentage of total articles (*n* = 33) reporting each variable. Numbers in red overlying bars represent percentage of patients describing each variable. Numbers in black within the bars represent the number of patients reporting each variable

**Fig. 2:**
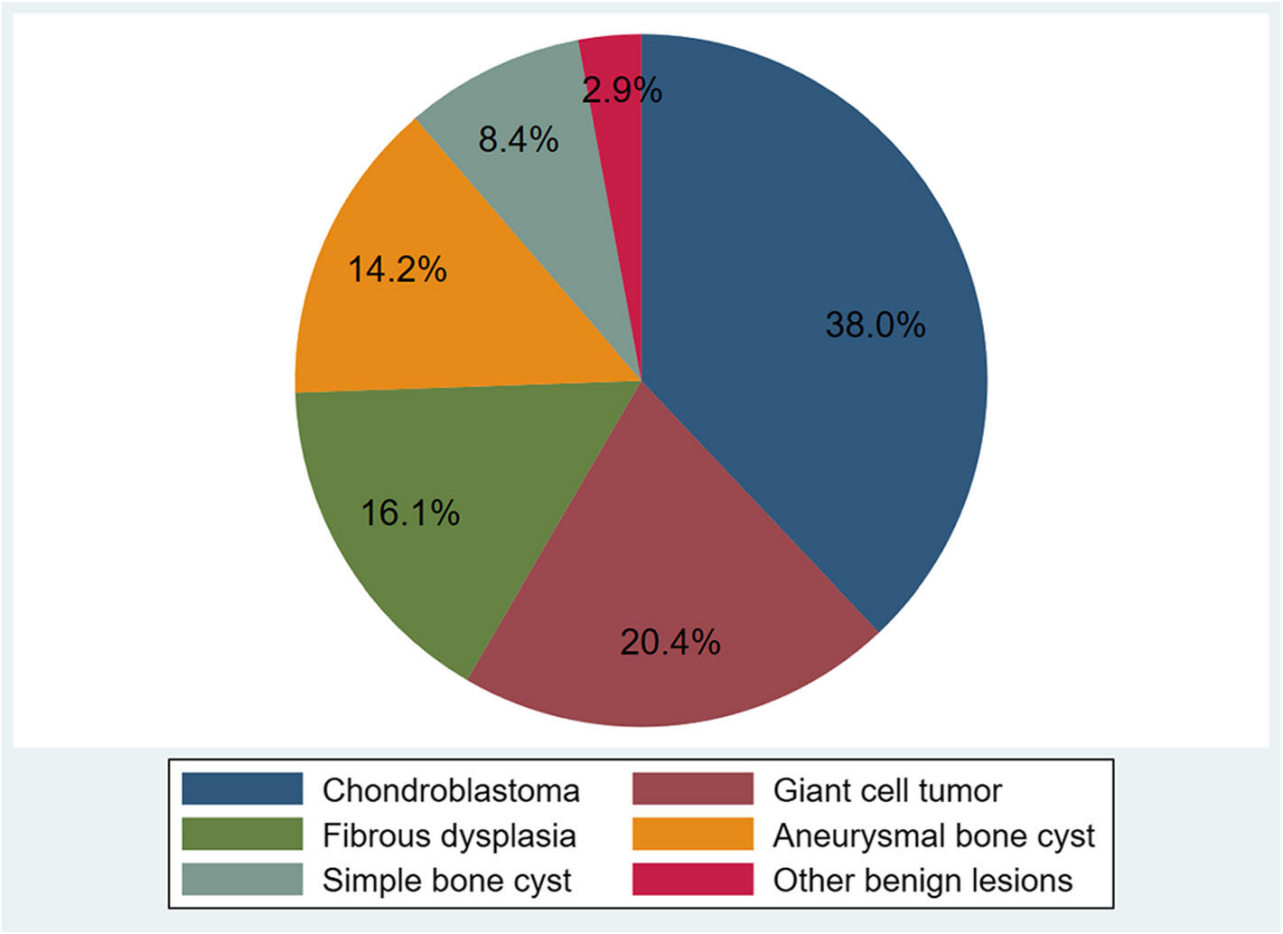
The pie graph shows tumor types

The initial search yielded 5523 results in PubMed (*n* = 1629), Embase (*n* = 3439) and Web of Science database (*n* = 455), respectively. Then we excluded articles which were not available in English (*n* = 777). After duplicates were removed (*n* = 1368), there were 3378 studies selected for title and abstract review and the resulting 189 articles for full-text screen. The references of articles which met the inclusion criteria were subjective to full-text review manually for potential eligibility. Ultimately, 33 articles [2, 4–14, 16, 17, 19, 23–40] including 274 patients fulfilled the inclusion criteria and were included in this systematic review (Table 1). The mean MINORS score was 8.45 (4–12), which showed a moderate quality of included studies. Patient age was reported in 30 studies and the average age was 21.2 (5–69) years (Fig. 1). For the 261 patients reported with age, 81 were children and 180 were adolescents or adults. Twenty-eight studies reported the gender of patients and on average 58.0% were male. All studies reported the diagnosis and surgical treatment. The most common diagnosis was CBT in 104 (38.0%), followed by GCT in 56 (20.4%) (Fig. 2). The size of lesion was reported in 10 studies, with a mean of 3.0 (0.8–5.5) cm. The duration of follow-up was reported in 31 studies, with a mean of 51.4 (4.0–234.0) months. Pathological fracture was present in 58 patients, with 6, 11, 19, 17 and 4 in CBT, GCT, aneurysmal bone cyst (ABC), fibrous dysplasia (FD) and simple bone cyst (SBC), respectively.

**Table 1:** Characteristics of included studies

Author	Year	Design	Patients (%)	Mean age (Years)	Tumor type	Surgery	Mean follow-up (Months)	MINORS score (quality)
Laitinen, M. K.et al.	2019	RC	14/177 (7.9%)	13.9	CBT	IC, RFA	NA	5/low
Luo, S.et al.	2019	CS	16/16 (100%)	9.5	ABC, SBC, Others	IC	24	10/moderate
Liu, Q.et al.	2019	CS	17/17 (100%)	16.3	CBT	IC	35.8	11/moderate
Panchwagh, Y.et al.	2018	CS	15/15 (100%)	26.1	GCT, FD, ABC, SBC	IC	77.2	10/moderate
Jamshidi, K.et al.	2018	CS	10/14 (71.4%)	8.8	SBC	IC	39.6	10/moderate
Rahman, M. A.et al.	2018	CS	1/16 (6.3%)	19	ABC	IC	60	8/low
Ozer, D.et al.	2018	CS	2/16 (12.5%)	13	CBT	IC	80	7/low
Farfalli, G. L.et al.	2017	RC	8/53 (15.1%)	NA	CBT	IC	78	12/high
Sharfman, Z. T.et al.	2016	CS	2/3 (66.7%)	24.5	Others	IC + arthroscope	16.5	6/low
Cho, H. S.et al.	2016	CS	2/7 (28.6%)	27.5	CBT, GCT	HR	48	9/moderate
Nishida, Y.et al.	2015	CS	8/8 (100%)	35.5	FD	IC	68.6	11/moderate
Nakamura, T.et al.	2015	CS	7/13 (53.8%)	33.2	GCT, FD, SBC	IC	82.2	8/low
Wijsbek, A. E.et al.	2014	CS	13/24 (54.2%)	27.6	GCT	IC, HR	77	11/moderate
Nisar, A.et al.	2014	CS	3/9 (33.3%)	20	CBT, FD	IC	27.3	8/low
Xu, H.et al.	2014	CS	13/14 (92.9%)	18	CBT	IC	66	10/moderate
Lalam, R. K.et al.	2014	CS	1/8 (12.5%)	19	CBT	RFA	14	9/moderate
Kundu, Z. S.et al.	2013	PC	16/16 (100%)	23.3	GCT, FD, ABC, Others	IC	32	11/moderate
Hu, Y. C.et al.	2012	CS	24/24 (100%)	34	CBT, GCT, ABC, FD	IC	40.1	7/low
Strong, D. P.et al.	2010	CS	10/10 (100%)	13.9	CBT	IC	64.8	9/moderate
Cho, H. S.et al.	2010	CS	12/12 (100%)	28.2	GCT	IC	58.3	10/moderate
Khalifa, Y. et al.	2010	CS	3/8 (37.5%)	23	ABC	IC	16.7	8/moderate
Sailhan, F.et al.	2009	RC	17/87 (19.5%))	12.5	CBT	IC	62.5	12/high
George, B.et al.	2008	CS	17/17 (100%)	16.4	GCT, FD, ABC, SBC	IC	34.3	8/low
Christie-Large, M.et al.	2008	CS	2/4 (50%)	13	CBT	RFA	13	6/low
Sakayama, K.et al.	2007	CS	6/10 (60%)	30.5	GCT	IC, HR	82	6/low
Petsas, T.et al.	2007	CS	2/2 (100%)	27	CBT	IC	12	5/low
Lin, P. P.et al.	2005	RC	4/48 (8.3%)	NA	CBT	IC	NA	8/low
Ozger, H.et al.	2003	CS	3/5 (60%)	18.7	ABC	IC	55.3	7/low
Wai, E. K.et al.	2001	CS	11/11 (100%)	23.6	GCT, FD, ABC	IC	50.7	11/moderate
Erickson, J. K.et al.	2001	CS	1/3 (33.3%)	26	CBT	RFA	48	6/low
Stricker, S. J.	1995	CS	3/3 (100%)	15.7	CBT	IC + arthroscope	24.7	4/low
Jaffe, K. A.et al.	1990	CS	7/7 (100%)	17	FD, ABC, SBC	IC	47	8/low
Tibrewal, S. B.	1986	CS	4/4 (100%)	22.5	GCT	IC	84	8/low

*CS* Case series, *RC* Retrospective cohort study, *PC* Prospective cohort study, *CBT* Chondroblastoma, *GCT* Giant cell tumor, *FD* Fibrous dysplasia, *ABC* Aneurysmal bone cyst, *SBC* Simple bone cyst, *IC* Intralesional curettage, *HR* Hip replacement, *RFA* Radiofrequency ablation, *NA* Not available, *MINORS* Methodological Index for non-Randomized Studies

In 104 CBT, there were 93 (90%) limited in the femoral head, only 11(10%) extended to the neck. The growth plate was open in 37 (35.6%), closed in 57 (54.8%) and not specified in 10 (9.6%). The distribution of 56 GCT in this region was head in 7(12.5%), neck in 10(17.9%) and head and neck in 39(69.6%), respectively (Table 2).

**Table 2:** Distribution of various lesions in the femoral head and neck

	CBT	GCT	FD	ABC	SBC	Others
Head	93	7	0	0	0	0
Neck	0	10	29	20	22	6
Head & Neck	11	39	15	19	1	2
Total	104	56	44	39	23	8

*CBT* Chondroblastoma, *GCT* Giant cell tumor, *FD* Fibrous dysplasia, *ABC* Aneurysmal bone cyst, *SBC* Simple bone cyst

### Surgical treatments

Intralesional curettage was applied in 257 (93.8%) patients, 5 of whom were under the assistance of arthroscopic visualization. Nine patients (1 CBT, 8 GCT) received wide resection with joint replacement as the initial treatment. RFA was utilized in 8 patients with CBT (Table 3).

**Table 3:** Surgery for different lesions

	CBT	GCT	FD	ABC	SBC	Others	Total
IC							
A	20	18	22	5	9	0	74
B	32	30	20	34	14	8	138
C	43	0	2	0	0	0	45
HR	1	8	0	0	0	0	9
RFA	8	0	0	0	0	0	8
Total	104	56	44	39	23	8	274

*IC* Intralesional curettage, *HR* Hip replacement, *RFA* Radiofrequency ablation, *A* Transcervical approach, *B* Open approach, *C* Direct approach, *CBT* Chondroblastoma, *GCT* Giant cell tumor of bone, *FD* Fibrous dysplasia, *ABC* Aneurysmal bone cyst, *SBC* Simple bone cyst

During intralesional curettage, adjuvants were applied in 189 (73.5%) patients, not applied in 41 (16.0%) patients and not specified in 27 (10.5%) patients. The adjuvant treatment protocol was as follows: high-speed burr in 116 patients or in combination with phenol or other toxic substances in 43 and 25 patients, respectively. The toxic substances were used alone in only 5 patients. Bone defect resulted from curettage was filled with bone graft in 205 (79.8%) patients, with cement in 8 (3.1%) patients and left empty in 7 (2.7%) patients. The other 37 (14.4%) patients were not specified.

### Functional outcome

For 9 patients received hip joint replacement, the MSTS score was 93.0% in median (80–100%) during a mean follow-up of 60.9 (21.0–85.0) months. The MSTS score was not reported in patients treated with RFA.

**Fig. 3:**
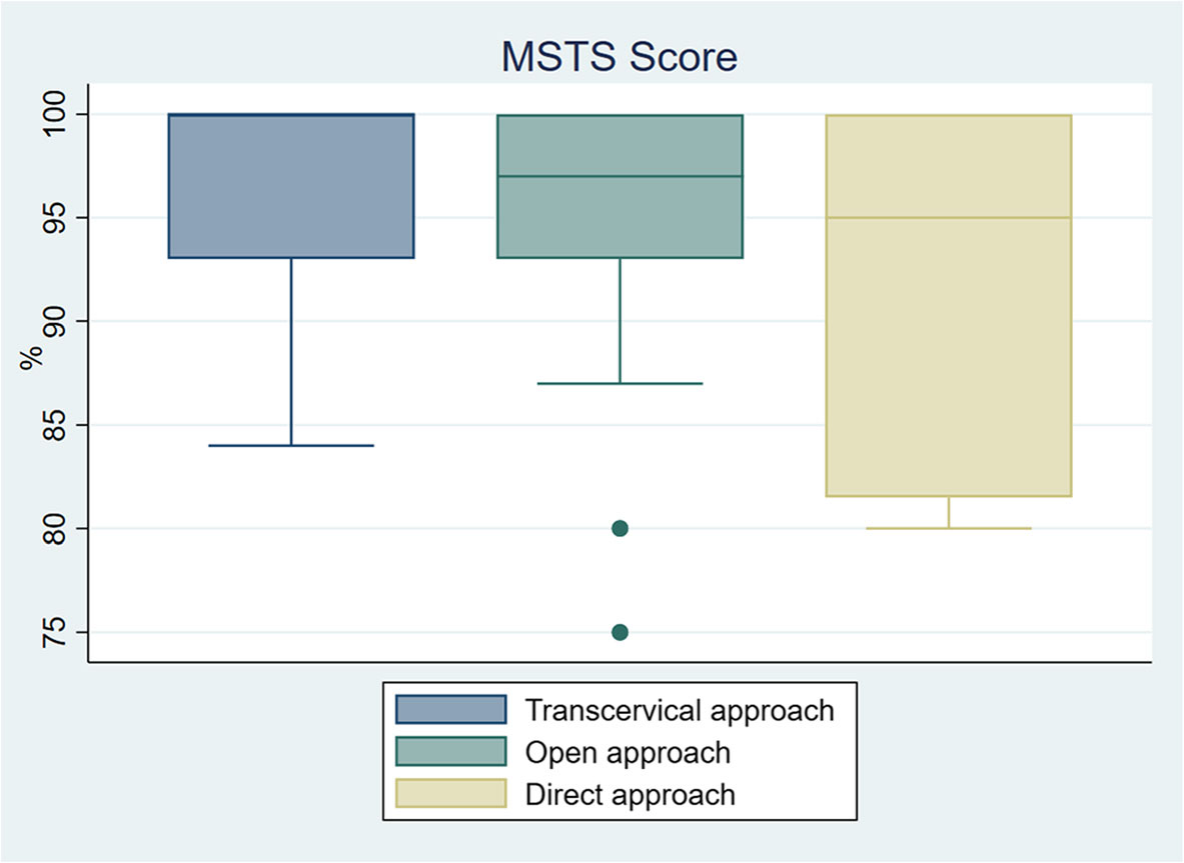
The box-plot graph shows MSTS score in different approaches for intralesional curettage

For the 257 patients treated with intralesional curettage, the MSTS score were reported in 149 patients, with a median of 97.0% (75.0–100.0%) during a mean follow-up of 54.0 (12.0–133.0) months. For extracapsular approach, the MSTS score were reported in 22 patients, with a median of 100.0% (84.0–100.0%) during a mean follow-up of 67.7 (12.0–128.0) months; for femoral neck fenestration, the MSTS score was reported in 87 patients, with a median of 97.0% (75.0–100.0%) during a mean follow-up of 49.3 (12.0–133.0) months; for articular trapdoor procedure, the MSTS score were reported in 40 patients, with a median of 95.0% (80.0–100.0%) during a mean follow-up of 56.5 (12.0–86.0) months. No difference was found in terms of MSTS score among patients who received the three types of intralesional curettage (*P* = 0.191, Kruskal-Wallis test) (Fig. 3).

### Local recurrence

Thirty-one patients (12.5%) experienced local recurrence following intralesional curettage during a mean follow-up of 51.5 (4.0–234.0) months (Table 4). The mean time interval from initial surgery to recurrence was 19.8 (4.0–180.0) months. The recurrence rate varied depending on the tumor type and the approaches for curettage. For intralesional curettage in 95 patients with CBT, in which all three surgical approaches were utilized, the recurrence rate was 35.0% (7/20) in transcervical approach group, followed by 12.5% (4/32) in open approach group and 2.3% (1/43) in direct approach group. The difference of recurrence rate among three approaches was significant, which was higher in the transcervical approach group (*P* < 0.001, Fisher’s Exact test). The recurrence rate was 29.7% (11/37) in patients with open growth plate, while only 1.7% (1/58) in those with a closed one. There is also existing a significant difference between the patients with and without open physis (*P* < 0.001, Fisher’s Exact test).

**Table 4:** Local recurrence and complications

	GD (immature with CBT)	AVN	DC	MF	IF	HO	CV	LC
A	3	2	0	1	1	0	0	16
B	0	0	0	4	3	1	3	14
C	0	2	6	0	0	1	0	1
Total	3	4	6	5	4	2	3	31

*A* Transcervical approach, *B* Open approach, *C* Direct approach, *GD* Growth disturbance, *AVN* Avascular necrosis of femoral head, *DC* Degenerative change, *MF* Mechanical failure, *IF* Infection, *HO* Heterotopic ossification, *CV* Coxa vara, *LC* Local recurrence

The highest recurrence rate after curettage was 33.3% (16/48) in patients with GCT. Hip replacement was performed in 8 patients with GCT primarily and associated with no recurrence. This difference was not statistically significant (*P* = 0.053, Fisher’s Exact test). GCT was associated with a high risk of pathological fracture (19.6%, 11/56) in this review. For the 11 patients with pathological fracture, 2 were treated with hip replacement at presentation. The other 9 were treated with curettage and the hip joint could be preserved in 8 patients (1 had local recurrence which was revised to hip replacement). As a result, the overall joint salvage rate for GCT in the femoral head and neck with pathological fracture was 72.7% (8/11). Although all 39 patients with ABC (19 with fracture) were curetted, there are only 1 local relapse occurred with the mean follow-up of 40 (6–72) months.

**Fig. 4:**
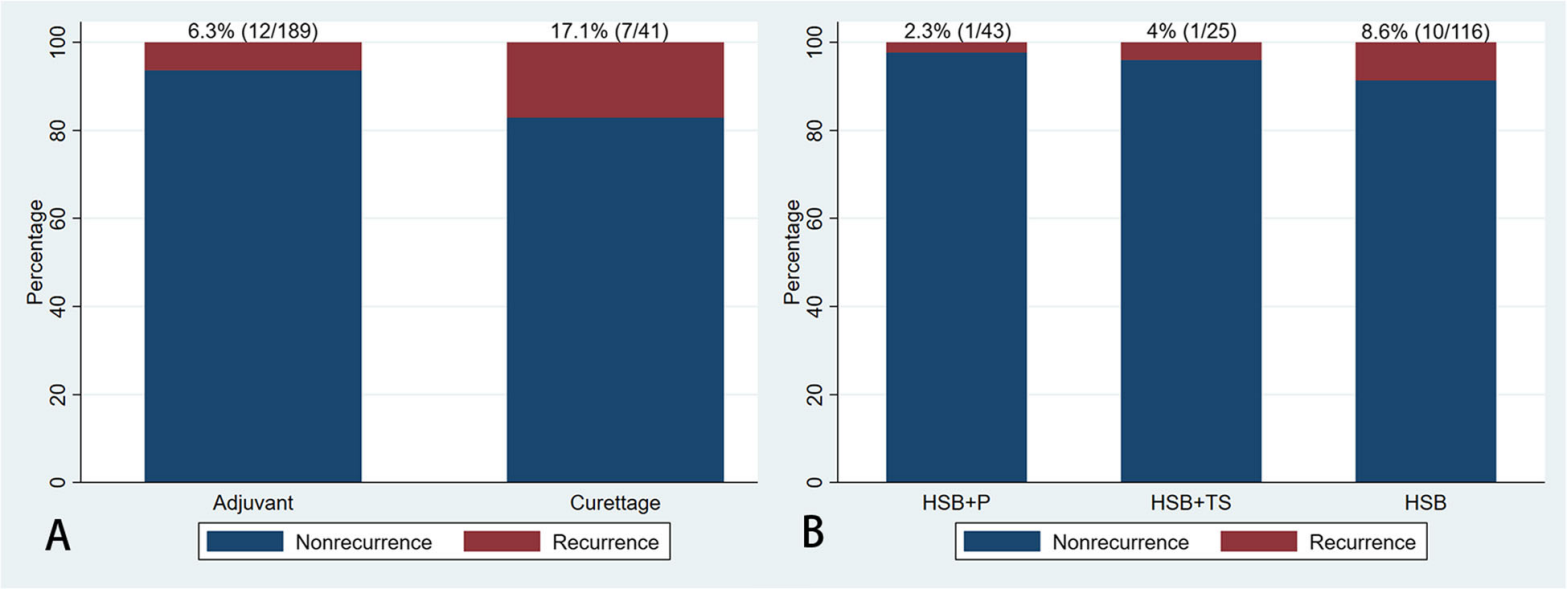
The bar graphs show the percentage of recurrence in relation to the adjuvant therapy; **A** shows the percentage of recurrence between groups with curettage only and with curettage combined with adjuvant therapy; **B** shows the percentage of recurrence among groups with different adjuvants. HSB: high-speed burr; HSB + P: high-speed burr+phenol; HSB + TS: high -speed burr+toxic substances

The use of different adjuvant therapies was evaluated. The local recurrence rate was 17.1% (7/41) in patients treated with curettage only and 6.3% (12/189) in those treated with curettage combined with adjuvants. Compared to the group with curettage only, the recurrence rate was found to be lower in the adjuvant group (*P* = 0.033, Fisher’s Exact test). While in the group with adjuvant therapy, the local recurrence rate was 8.6% (10/116) in patients treated with high-speed burr only, 2.3% (1/43) in those with high-speed burr plus phenol and 4% (1/25) in those with high-speed burr with other toxic substances (Fig. 4). Additional utilization of phenol and other toxic substances did not further decrease the rate of recurrence (*P* = 0.424, Fisher’s Exact test).

There was only 1 local recurrence each in 39 ABC, 44 FD and 23 SBC, respectively. Among the patients who experienced recurrence, further treatment was performed to reach local control for 29 patients, while the other 2 were kept conservatively (1 FD and 1 SBC). In 10 patients (all were GCT), hip replacement was chosen as a revision surgery while the other 19 patients (12 CBT, 6 GCT and 1 ABC) were treated with re-curettage. No patient was reported to develop tumor relapse following joint replacement and RFA at a mean follow-up time of 60.9 (21.0–85.0) months and 22.0 (12.0–48.0) months, respectively.

### Other complications

Besides local recurrence, there were other 27 (10.5%) important complications reported in 257 patients following intralesional curettage (Table 4). Growth disturbance, which theoretically could only occur in skeletally immature patients, was reported in 3 of 37 CBT with open growth plate. All of these patients were treated with the transcervical approach. Two patients each developed AVN in the transcervical and direct approach group. Only two articles mentioned 6 of 25 patients who developed degenerative changes of hip joint after the tumor cured, and these 6 patients were all treated with direct approach. There were also wound infection in 4, mechanical failure in 5, heterotopic ossification in 2 and coxa vara deformity in 3 reported. No malignant transformation and metastasis were reported in these 274 patients.

## Discussion

The surgical management of benign osteolytic lesions in the femoral head and neck is not clear, and in many cases depends on the experience of the surgeon, the lesion size and location, the extent of bone destruction and the age of the patients. Although previous scattered studies have reported reasonable results from treating benign bone tumors and tumor-like lesions of this site, no systematic review to date has investigated the optimal option of surgical treatment and their outcomes.

With the available patients selected, the lesions consist of CBT, GCT, FD, ABC, SBC and others in a descending order, which is in line with Jaffe et al. [12]. Incidental finding is rare, unremitting hip pain or limp are the main cause for clinical consultation. Furthermore, the marked bony destruction renders this area at risk for a pathological fracture. In this review, 57 patients (20.8%) came to hospital with established fracture.

Intralesional curettage is the most common option due to its preservation of the hip joint and a much better postoperative function [33]. Three approaches were used in 257 patients treated with curettage. Gaining surgical access through the femoral neck to the femoral head in CBT resulted in significant high local recurrence than the other two approaches, as we can see from our results. This is not surprising, because, technically, you have to curette a cavity at the end of a long thin passage through a drill hole created up the femoral neck, which is impossible to provide a clear visualization of the whole lesion even under image intensifier control or assisted by arthroscopy [27, 29]. Strong et al. [11] reported ten patients with femoral head CBT. Two of five patients treated with curettage via transcervical approach developed local recurrence, while no recurrence was encountered in another five patients with direct approach. The status of physis also plays a role in the local recurrence after curettage in CBT. The patients with open physis had a higher incidence of recurrence, which is in consistent with Suneja’s point [3]. The author reported 53 patients with CBT treated with intralesional curettage and younger patients were more likely to develop local recurrence. The explanation is that less extensive curettage may be performed in the younger patients due to concerns about the growth plate.

The incidence of recurrence after curettage was higher in patients with GCT of proximal femur than those with lesions located at other anatomical sites. The recurrence rate of GCT in this pooled analysis is 33.3% (16/48), which is in the range of previous studies [5, 28, 41]. With the limited number of patients, we could not confirm that joint replacement with wide resection provides a better local control in terms of oncological outcome, although it is thought to be beneficial by some researchers [42, 43]. On the other hand, pathological fracture complicates the cases as surgeons must decide that whether joint preserving surgery should be performed. Sakayama et al. [26] recommend that hip replacement should be avoided whenever possible, even the functional and oncological outcome of this group is satisfied, while Wijsbek et al. [5] advocated hip replacement as primary procedure. Overall, 8 of 11 patients with GCT presenting pathological fracture could preserve their native joint in this systematic review.

The reported rate of local recurrence of ABC varied considerably, ranging from 10 to 30% [44, 45]. However, very low local recurrence rate has been confirmed in recently published series. Wang et al. [46] reported 31 ABC (45% with pathological fracture) treated with curettage combined with high-speed burring, only 1 patient had local recurrence at a mean follow-up of 7 years. Rahman et al. [33] reported 16 patients with proximal femur ABC (25% with pathological fracture) treated with extended curettage and cryosurgery, there was also only 1 patient with pathological fracture developing local recurrence at a mean follow-up of 50.5 months. All of these results were in agreement with our finding.

The utilization of high-speed burr with or without phenol or other toxic substances has been accepted widely for achieving extensive surgical margin after intralesional curettage of benign bone tumors [43]. Klenke et al. [47] investigated the efficacy of phenol in the treatment of GCT. In their study, 95 out of 118 patients were treated with intralesional surgery. Curettage, high-speed burr and phenol therapy was performed in 40 patients while the others were under the same treating condition except for phenol use. The author found that there was no improvement in recurrence-free survival with local phenol use. The same result was observed by Arbeit et al. [43]. Our findings correspond well to these authors’ researches, which emphasized the significance of performing more thorough curettage instead of multiple adjuvants in decreasing recurrence.

We compared functional outcome among different methods of curettage and found that all these three curettage strategies result in satisfactory function outcome evaluated by MSTS score. In the present review, only 1 article reported the patient-completed outcome with Short Form-36 (SF-36) [31]. We recommend future studies in assessment of functional outcome, using a more patient-reported one, are required to establish the superiority of certain surgical treatment.

In terms of local complications, the growth disturbance in skeletally immature patients poses therapeutic dilemmas to surgeons when treating femoral head lesions. However, this complication was not frequently encountered among patients with growth remaining after curettage. In this review, only 3 patients with CBT experienced this complication following curettage with transcervical approach. Some authors pointed that the epiphysis fuses at the age of about 15 years in males and 14 in females, so breaching this in children near skeletal maturity is not likely to cause significant growth arrest [11]. In addition, some authors agreed that the proximal femoral physis only contributes a little proportion of growth and most of growth in femur occurs in the distal physis, so this inequality may not be clinically significant.

Surgical femoral head dislocation could provide a better local control, while this method has a limitation that the size of trapdoor must be less than 30% of the femoral head surface otherwise a premature arthritis may be expected [15, 48]. Farfalli et al. [4] shared the experience in treating femoral head chondroblastoma in 8 patients, as a result, degenerative change was detected in 5 of them and 4 patients had hip replacement to achieve pain relief. Another question also rises whether femoral head dislocation could result in a higher incidence of AVN as time passing by. It is believed that gaining surgical access to the proximal epiphysis of the femur may compromise the blood supply to the femoral head especially after dislocation. Totally, 2 patients with CBT in the femoral head developed AVN with direct approach, while the other 2 patients did with transcervical approach, which is caused by pathological fracture in 1 with ABC and not specified in another 1 with SBC. Ganz et al. [49] introduced in detail the method of surgical dislocation to resolve hip diseases. None of 213 patients undergoing surgical dislocation developed AVN of femoral head. This result demonstrates the safety of this procedure and should be more advocated in dealing with femoral head lesions.

This systematic review was subject to several limitations. First, although three literature databases have been used, we might have missed possible relevant publications that are not listed in these libraries. Second, most of the studies included were case series with small sample size due to rarity of this situation, which inevitably weakened the level of evidence of this review. Additionally, the studies comprising our review were primarily retrospective and uncontrolled design, thus prone to selection and observer bias. Third, the surgical techniques used within intralesional curettage varied according to the types of lesion. Probably, the experience of surgeons, postoperative managements and rehabilitation regimens varied as well and further confounded the outcomes. Even considering these shortcomings, the systematic review described here may serve as a framework which may help the surgeon to determine the optimal surgical methods for benign osteolytic lesions in the femoral head and neck.

## Conclusion

The majority of benign osteolytic lesions in the femoral head and neck can be treated with intralesional curettage with acceptable local tumor control and satisfactory function. The incidence of local recurrence might be decreased dramatically for lesion access under direct visualization. The native joint maintenance could be achieved even in patients with aggressive lesions presenting pathological fracture.

## Supplementary Information

**Additional file 1: Figure S1.** The flowchart shows steps for literatures search and selection.

## Data Availability

The datasets used and/or analyzed during the current study available from the corresponding author on reasonable request.
