# Supplementary figures and images for: Surgical treatment of benign osteolytic lesions in the femoral head and neck: a systematic review
Source: BMC Musculoskelet Disord. 2021 Jun 16;22:549. doi: 10.1186/s12891-021-04442-y (PMC8210383; doi:10.1186/s12891-021-04442-y)

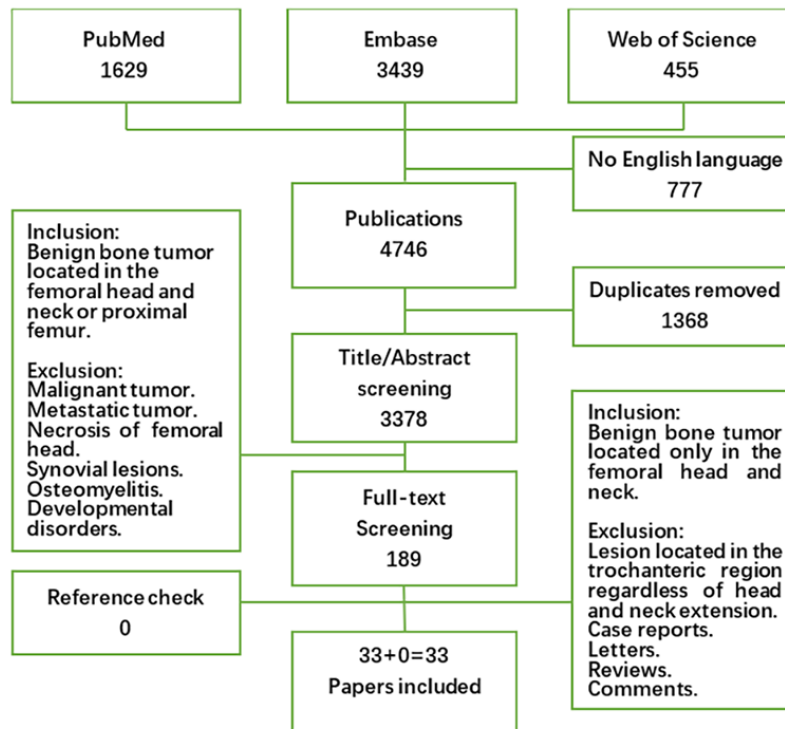

Figure S1. The flowchart shows steps for literatures search and selection.

Supplement: Supplementary file 1 — Additional file 1: Figure S1. The flowchart shows steps for literatures search and selection. [file 12891_2021_4442_MOESM1_ESM.pdf]
